# High Brain Ammonia Tolerance and Down-Regulation of Na^+^:K^+^:2Cl^-^ Cotransporter 1b mRNA and Protein Expression in the Brain of the Swamp Eel, *Monopterus* *albus*, Exposed to Environmental Ammonia or Terrestrial Conditions

**DOI:** 10.1371/journal.pone.0069512

**Published:** 2013-09-19

**Authors:** Yuen K. Ip, Zhisheng Hou, Xiu L. Chen, Jasmine L. Y. Ong, You R. Chng, Biyun Ching, Kum C. Hiong, Shit F. Chew

**Affiliations:** 1 Department of Biological Sciences, National University of Singapore, Singapore, Republic of Singapore; 2 Natural Sciences and Science Education, National Institute of Education, Nanyang Technological University, Singapore, Republic of Singapore; Universitat de Barcelona, Spain

## Abstract

Na^+^:K^+^:2Cl^-^ cotransporter 1 (NKCC1) has been implicated in mediating ischemia-, trauma- or ammonia-induced astrocyte swelling/brain edema in mammals. This study aimed to determine the effects of ammonia or terrestrial exposure on ammonia concentrations in the plasma and brain, and the mRNA expression and protein abundance of *nkcc*/Nkcc in the brain, of the swamp eel *Monopterus* *albus* . Ammonia exposure led to a greater increase in the ammonia concentration in the brain of *M. albus* than terrestrial exposure. The brain ammonia concentration of *M. albus* reached 4.5 µmol g^-1^ and 2.7 µmol g^-1^ after 6 days of exposure to 50 mmol l^-1^ NH_4_Cl and terrestrial conditions, respectively. The full cDNA coding sequence of *nkcc1b* from *M. albus* brain comprised 3276 bp and coded for 1092 amino acids with an estimated molecular mass of 119.6 kDa. A molecular characterization indicated that it could be activated through phosphorylation and/or glycosylation by osmotic and/or oxidative stresses. Ammonia exposure for 1 day or 6 days led to significant decreases in the *nkcc1b* mRNA expression and Nkcc1b protein abundance in the brain of *M. albus*. In comparison, a significant decrease in *nkcc1b* mRNA expression was observed in the brain of *M. albus* only after 6 days of terrestrial exposure, but both 1 day and 6 days of terrestrial exposure resulted in significant decreases in the protein abundance of Nkcc1b. These results are novel because it has been established in mammals that ammonia up-regulates NKCC1 expression in astrocytes and NKCC1 plays an important role in ammonia-induced astrocyte swelling and brain edema. By contrast, our results indicate for the first time that *M. albus* is able to down-regulate the mRNA and protein expression of *nkcc1b*/Nkcc1b in the brain when confronted with ammonia toxicity, which could be one of the contributing factors to its extraordinarily high brain ammonia tolerance.

## Introduction

Animals cannot store excess amino acids. Therefore dietary amino acids in excess of the amounts needed for growth and maintenance of protein turnover are preferentially degraded over carbohydrates and lipids in the liver [1]. In vertebrates, amino acids are degraded mainly through transdeamination in the liver, during which the α-amino group is released as ammonia in the form of NH_4_^+^. In aqueous solution, ammonia can be present as gaseous NH_3_ and cationic NH_4_^+^, the ratio of which is pH dependent. Ammonia is toxic for many reasons. The most acute effects of ammonia probably arise from the ability of NH_4_^+^ to substitute for K^+^ to activate ion transporters [2] and disrupt electrochemical gradients in the central nervous system [3]. Several theories (glutamatergic dysfunction, glutamine accumulation leading to astrocyte swelling, activation of *N*-methyl-ᴅ-aspartate-type glutamate receptors) have been proposed for the mechanisms of acute ammonia toxicity in mammalian brains [4,5]. Excessive activation of *N*-methyl-ᴅ-aspartate-type glutamate receptors by ammonia [6,7] is neurotoxic, leading to oxidative stress, neuronal degeneration and death [8]. More recent studies in mammals have implicated the involvement of oxidative stress and mitochondrial permeability transition in ammonia neurotoxicity [9,10]. The mitochondrial permeability transition could result from the permeation of cytosolic glutamine through the inner mitochondrial membrane into mitochondria, and the subsequent release of ammonia from glutamine catalyzed by glutaminase in the mitochondrial matrix of astrocytes [11]. However, all these theories of mechanisms of ammonia toxicity have yet to be confirmed in fish brain [12,13,14].

Aquatic fishes keep body ammonia levels low by excreting excess ammonia mainly as NH_3_ across the body surface, usually the gills. Air-breathing is one of several adaptive responses utilized by fishes dwelling in habitats where O_2_ supplies may be severely depleted [15]. Air-breathing fishes have modified gill morphology and morphometry, with or without accessory breathing organs, which may render branchial ammonia excretion inefficient. While most air-breathing fishes remain aquatic, some have evolved to emerge from water, make excursions onto land, or even burrow into mud when the external media dry up. When a fish is out of water, it is confronted with problems of ammonia excretion because of a lack of water to flush the branchial and cutaneous surfaces. Some air-breathing fishes can be trapped in puddles of water occasionally or in crevices for many days, during which the continual excretion of endogenous ammonia into a small volume of external medium would lead to high external ammonia concentrations. In high concentrations of environmental ammonia, fishes are confronted simultaneously with endogenous ammonia retention and exogenous ammonia uptake. Therefore, air-breathing fishes, especially amphibious ones, are equipped with various strategies to ameliorate ammonia toxicity during emersion or exposure to environmental ammonia [14,16,17,18,19,20].

The swamp eel, *Monopterus* *albus* (Zuiew), belongs to Order Synbranchiformes, Family Synbranchidae, and is commonly found in muddy ponds, swamps, canals and rice fields in India, Malaysia, Indonesia and Southern China. It is an obligate air breather with vastly atrophied gills, which become a skin fold within the opercular chamber. As an air breather, *M. albus* can survive on land for an extended period. Since no water is available to flush the branchial or cutaneous surfaces during emersion, ammonia excretion becomes inefficient leading to significant increases in ammonia concentrations in the body. After 72 h of terrestrial exposure, ammonia concentrations in the liver, brain and plasma of *M. albus* increased by 3-fold, 3.5-fold and 5-fold, respectively, as compared to those of the control kept in freshwater [21]. In the muscle and gut, the ammonia concentration reached the highest level of 6.9 µmol g^-1^ and 4.5 µmol g^-1^, respectively, after 6 days of terrestrial exposure [21]. The high tolerance to ammonia at the cellular and tissue levels contributes partially to the extremely high tolerance of *M. albus* to environmental ammonia [22]. After 6 days of exposure to 75 mmol l^-1^ NH_4_Cl at pH 7.0, the ammonia concentrations in the muscle, liver, brain, and gut of *M. albus* reach 11.5, 15.2, 6.5, and 7.5 µmol g^-1^, respectively. Simultaneously, the plasma ammonia concentration increases to 3.5 mmol l^-1^, which would presumably reduce the magnitude of the inwardly-directed NH_3_/NH_4_^+^ gradients and lessen the net influx of exogenous ammonia. *Monopterus* *albus* can also survive a high sub-lethal dose (10 µmol g^-1^ fish) of intraperitoneal injection with CH_3_COONH_4_ [23].

Since the blood brain barrier permeability for ^13^NH_4_^+^ is only ~0.5% that of ^13^NH_3_ in Rhesus monkey [24], the traditional assumption is that NH_3_ can pass through the blood-brain barrier by diffusion, and NH_4_^+^ translocation can be neglected [3]. However, effects of pH on ammonia uptake are often less pronounced than expected, although they are in the direction predicted by the NH_3_ diffusion hypothesis. Therefore, it has been proposed that NH_4_^+^ can also permeate the blood-brain barrier with the possible involvement of bumetanide-inhibitable Na^+^:K^+^:2Cl^-^ cotransporter (NKCC), barium-inhibitable K^+^ channel, Na^+^/K^+^-ATPase and Rhesus glycoproteins [25]. Once NH_3_ and NH_4_^+^ get through the blood-brain barrier, they can permeate the plasma membrane of neurons and astrocytes through various transport systems, including those ion channels, exchangers, and transporters essential for cell volume regulation [26,27]. Thus, ammonia-induced functional changes in these transport systems would result in alterations of ion and water homeostasis [28].

The electroneutral NKCC is present in a wide variety of animal cells and tissues [29]. Two isoforms of NKCC, NKCC1 and NKCC2, have been identified [30]. In mammals, NKCC1 is present in many cell types, including astrocytes, neurons and oligodendrocytes [31,32], while NKCC2 is localized exclusively to the kidney [33]. NKCC transports Na^+^, K^+^, and 2Cl^-^ into cells under both physiological and pathophysiological conditions and can be inhibited by either bumetanide or furosemide [29]. It is involved in ion transport across secretory and absorptive epithelia [29], NH_4_^+^ transport [34], and the maintenance and regulation of cell volume and ion gradients [35]. In states of dehydration, the transport of ions and obligated water molecules into the cell through NKCC restores cell volume. However, inappropriate activation of NKCC would lead to cell swelling and tissue edema. NKCC1, in particular, has been shown to play an important role in the mediation of ischemia- or trauma-induced astrocyte swelling/brain edema in mammals [27]. Recent studies suggest that NKCC1 activation is also involved in ammonia-induced astrocyte swelling/brain edema caused by thioacetamide-induced acute liver failure [36]. Therefore, this study was undertaken to obtain the cDNA coding sequence of *nkcc1* from the brain of *M. albus*, and to examine the effects of 1 day or 6 days of exposure to environmental ammonia (50 mmol l^-1^ NH_4_Cl in freshwater) or terrestrial conditions on its mRNA expression and protein abundance in the brain. The hypothesis tested was that *M. albus* had the ability to down-regulate the expression of *nkcc1*/Nkcc1 in its brain in response to the accumulation of ammonia in the blood and brain during ammonia or terrestrial exposure, which contributed in part to its high brain ammonia tolerance.

## Materials and Methods

### Fish

Specimens of *M. albus* (150–250 g) were purchased from a local fish distributor in Singapore. Fish were maintained in plastic tanks in freshwater at 25°C under a 12 h: 12h dark: light regime. No aeration was provided because *M. albus* is an obligatory air-breather. No attempt was made to separate the sexes. Fish were acclimated to laboratory conditions for at least 1 week before experimentation, during which they were fed fish meat once every two days. Food was withheld during the experimental period. Approval to undertake this study (IACUC 021/10A and 098/10) was obtained from the Institutional Animal Care and Use Committee of the National University of Singapore.

### Experimental conditions and collection of samples

Control fish (total *N*=13; *N*=5 each for ammonia assay and molecular work, and *N*=3 for Western blot) were immersed in 25 volumes (v/w) of freshwater in plastic tanks with free access to air. Fish subjected to ammonia exposure were immersed in freshwater containing 50 mmol l^-1^ NH_4_Cl (pH 7.0), for either 1 day or 6 days (total *N*=13 for each group), with daily changes of NH_4_Cl solution. Fish were killed at the end of day 1 or day 6 of ammonia exposure. Control fish and fish exposed to ammonia were killed with an overdose of neutralized MS-222 (0.2%) followed with a strong blow to the head. For exposure to terrestrial conditions, fish were kept in plastic aquaria tanks (50 cm length x 30 cm width x 10 cm height) containing a thin film (100 ml) of freshwater for either 1 day or 6 days (total *N*=13 for each group). Water was replenished daily and experimental fish killed at the end of day 1 or day 6. Fish exposed to terrestrial conditions were killed with a strong blow to the head. Blood was collected from the severed caudal artery into sodium heparin-coated capillary tubes. The collected blood was centrifuged at 4000 ×g at 4°C for 10 min to obtain the plasma. The plasma was deproteinized in an equal volume (v/v) of ice-cold 6% trichloroacetic acid (TCA) and centrifuged at 10,000 ×g at 4°C for 15 min. The resulting supernatant was kept at -80°C for analysis of ammonia. The whole brain from an individual fish was quickly excised from both control and experimental fish, frozen in liquid nitrogen and stored at -80°C until ammonia analysis.

### Determination of ammonia concentrations in the brain and plasma

The frozen brain samples were weighed, ground to a powder in liquid nitrogen, and homogenized three times in 5 volumes (w/v) of ice-cold 6% TCA at 24,000 rpm for 20 s each using an Ultra-Turrax homogenizer with intervals of 10 s between each homogenization. The homogenate was centrifuged at 10,000 ×g at 4°C for 30 min to obtain the supernatant. The pH of the supernatant obtained was adjusted to between 6.0 and 6.5 with 2 mol l^-1^ KHCO_3_, and the ammonia concentration was determined according to the method of Bergmeyer and Beutler [37]. Results were expressed as μmol g^-1^ wet mass tissue or mmol ml^-1^ plasma.

### Total RNA extraction and cDNA synthesis

The total RNA was extracted from brain samples using Tri Reagent^TM^ (Sigma-Aldrich Co., St. Louis, MO, USA) and further purified using the RNeasy Plus Mini Kit (Qiagen GmbH, Hilden, Germany). After extraction, RNA was quantified spectrophotometrically using a Hellma TrayCell (Hellma GmbH & Co. KG, Müllheim, Germany) and checked electrophoretically to verify the RNA integrity. The total RNA (1 µg) isolated from the brain of *M. albus* was reverse transcribed into first strand cDNA using oligo (dT)_18_ primers and the RevertAid^TM^ First Strand cDNA synthesis kit (Thermo Fisher Scientific Inc., Waltham, MA, USA) following the manufacturer’s protocol.

### Polymerase chain reaction (PCR) and cloning

The partial *nkcc1* sequence was obtained using the primers (Forward: 5’-CGC TGY ATG CTV AAT ATC TGG-3’; Reverse: 5’-CGC TGY ATG CTV AAT ATC TGG-3’) designed from the highly conserved regions based on multiple alignments of the *nkcc1* sequences from various fish species available in the Genbank (http://www.ncbi.nlm.nih.gov/Genbank). PCR was carried out in a Bio-Rad Peltier thermal cycler (Bio-Rad Laboratories, Hercules, CA, USA) using Dreamtaq^TM^ DNA polymerase (Thermo Fisher Scientific Inc.) under the following cycling conditions: 95°C (3 min), followed by 35 cycles of 95°C (30 s), 55°C (30 s), 72°C (2 min) and a final cycle of extension at 72°C (10 min). The PCR product was separated by electrophoresis in 1% agarose gel. A band of the estimated size was excised and purified from the gel using FavorPrep^TM^ Gel Purification Mini Kit (Favorgen Biotech Corp., Ping-Tung, Taiwan) according to the manufacturer’s protocol. Purified PCR products were ligated into pGEM-T easy vector (Promega Corporation, Madison, WI, USA), transformed into JM109 *Escherichia coli* competent cells and plated onto Luria-Bertani (LB) agar with ampicillin, IPTG and X-gal. Colony-PCR was performed on selected white colonies. Colonies with insert of estimated size were grown overnight in LB/ampicillin broth in a shaking incubator (37°C, 250 rpm). Plasmid extraction was performed using AxyPrep^TM^ Plasmid Miniprep Kit (Axygen Biosciences, Union City, CA, USA). Multiple clones of each fragment were sequenced bidirectionally by cycle sequencing using BigDye^®^ Terminator v3.1 Cycle Sequencing Kit (Life Technologies Corporation, Carlsbad, CA, USA), and subsequently purified by ethanol/sodium acetate precipitation. Purified products were automatically sequenced using the 3130XL Genetic Analyzer (Life Technologies Corporation). The fragments were verified to be *nkcc1* from GenBank database.

### Rapid amplification of cDNA ends (RACE)

Total RNA (1 µg) isolated from the brain of *M. albus* was reverse transcribed into 5’-RACE-Ready cDNA and 3’-RACE-ready cDNA using the SMARTer^TM^ RACE cDNA Amplification kit (Clontech Laboratories, Mountain View, CA, USA). RACE-PCR was performed using Advantage^®^ 2 PCR kit (Clontech Laboratories) to generate the 5’ and 3’ cDNA fragments. RACE primers (Forward: 5’-AGA CAT CAA CAC CAA ACC CAA GAA A-3’; Reverse: 5’-TGT ACG GCT CGA TCA GCT CCT TA-3’) were designed based on the partial cDNA sequences obtained. RACE-PCR cycling conditions were: 25 cycles of 94°C (30 s), 68°C (30 s) and 72°C (4 min). RACE-PCR products were separated using gel electrophoresis, purified and sequenced. Sequence assembly and analysis were performed using BioEdit version 7.1.11 [38].

### Deduced amino acid sequence and phylogenetic analysis

The nucleotide sequence of *nkcc* obtained from the brain of *M. albus* was translated into a putative amino acid sequence using ExPASy Proteomic server (http://web.expasy.org/translate/). The deduced amino acid sequence was aligned and compared with selected Nkcc1/NKCC1 sequences from various animal species using BioEdit. The sequence identity matrix generated was used to confirm the identity of the Nkcc1 from *M. albus*. Transmembrane domains were identified using MEMSATS and MEMSAT-SVA provided by PSIPRED protein structure prediction server (http://bioinf.cs.ucl.ac.uk/psipred/) [39]. Potential phosphorylation sites were predicted using NetPhos 2.0. Multiple sequence alignments using amino acid sequences from selected species were also performed using ClustalX2.

Amino acid sequences of Nkcc1/NKCC1 from other animals were obtained from Genbank of UniProtKB/TrEMBL with the following accession numbers: *Anabas* *testudineus* Nkcc1a (AFK29496.1), *Anguilla* *anguilla* Nkcc1a (CAD31111.1), *Anguilla* *anguilla* Nkcc1b (CAD31112.1), *Bos taurus* NKCC1 (NP_777207.1), *Danio rerio* Nkcc1 (NP_001157126.1), *Dicentrarchus* *labrax* Nkcc1 (ABB84251.1), *Homo sapiens* NKCC1 (P55011.1), *Homo sapiens* NKCC2 (NP_000329.2), *Macaca mulatta* NKCC1 (NP_001248714.1), *Oreochromis* *mossambicus* Nkcc1a (AAR97731.1), *Oreochromis* *mossambicus* Nkcc1b (AAR97732.1), *Oreochromis* *mossambicus* Nkcc2 (AAR97733.1), *Oryctolagus cuniculus* NKCC2 (NP_001164442.1), *Rattus norvegicus* NKCC1 (NP_113986.1), *Rattus norvegicus* NKCC2 (NP_001257547.1), *Sarotherodon* *melanotheron* Nkcc1 (ACY05529.1), *Squalus* *acanthias* Nkcc1 (AAB60617.1), *Takifugu* *obscurus* Nkcc2 (BAH20440.1), *Xenopus laevis* NKCC1 (ABN05233.1), and *Strongylocentrotus purpuratus* Nkcc (NP_001106707.1; as an outgroup for phylogenetic analysis). A complete alignment was performed for the deduced amino acid sequence of Nkcc from the brain of *M. albus* and those of other animals using ClustalW. The percentage similarity was then computed using the "sequence identity matrix" function provided in BioEdit. Phylogenetic analysis was done using neighbor-joining method with 100 bootstrap replicates with Phylip [40].

### Quantitative real-time PCR (qPCR)

Samples of total RNA from brains of *M. albus* were processed through a genomic DNA eliminator spin column from the Qiagen RNeasy Plus Mini Kit (Qiagen) to remove genomic DNA. The total RNA (1 µg) was reverse transcribed using random hexamer primers with RevertAid^TM^ first strand cDNA synthesis kit (Thermo Fisher Scientific Inc.). qPCR was performed in triplicates using a StepOnePlus™ Real-Time PCR System (Life Technologies Corporation). The standard cDNA (template) was serially diluted (from 10^10^ to 10^2^ specific copies per 2 µl). The PCR reactions contained 5 µl of KAPA SYBR^®^ FAST Master Mix (2X) ABI Prism® (Kapa Biosystems, Woburn, MA, USA), with 0.2 µl each of primers (Forward: 5’-AGG CTC TGT GTA AGG ACA A-3’; Reverse: 5’-ATG GGA GCA ATG ATG TTC AG-3’) and 2 µl of cDNA (equivalent to 1 ng of RNA) or standard in a total volume of 10 µl.

Cycling conditions were 95°C (20 s), followed by 40 cycles of 95°C (3 s) and 60°C (30 s). Data (threshold cycle as C_T_ values) were collected at each elongation step. Melt curve analysis was performed after runs by increasing from 60°C to 95°C in 0.3°C increments to confirm the presence of only a single product. The PCR products were separated in a 2% agarose gel to verify the presence of a single band.

To determine the absolute quantity of *nkcc1b* transcripts in a qPCR reaction, a pure amplicon (standard) of a defined region of cDNA (151 bp) was obtained from the brain of *M. albus* according to the methods of Gerwick et al. [41]. PCR was performed using qPCR primers and cDNA as a template in a final volume of 25 µl with the following cycling conditions: initial denaturation of 95°C (3 min), followed by 35 cycles of 95°C (30 s), 60°C (30 s) and 72°C (30 s) and 1 cycle of final extension of 72°C (10 min). The PCR product was separated in a 2% agarose gel, and the band of appropriate size excised and purified using FavorPrep^TM^ Gel Purification Mini Kit (Favorgen Biotech Corp.). The purified product was cloned using pGEM^®^-T Easy vector (Promega Corporation) and the presence of the insert in the recombinant clones was confirmed by sequencing. The cloned circular plasmid was quantified using a spectrophotometer. A standard curve was obtained by plotting the natural log of concentration (copies per μl) on the Y axis and threshold cycle (C_T_) on the X axis. The C_T_ slope, PCR efficiency, *Y* intercept and correlation coefficient (*R* ^2^) were calculated using the default settings of StepOne^TM^ Software v2.1 (Life Technologies Corporation). Diluted standards were stored at -20°C. The amplification efficiency was 93.1%. The quantity of transcript in an unknown sample was determined from the linear regression line derived from the standard curve and expressed as copies of transcripts per ng cDNA.

### SDS-PAGE and Western blotting

Brain samples were homogenized three times in five volumes (w/v) of ice cold buffer containing 50 mmol l^-1^ Tris HCl, (pH 7.4), 1 mmol l^-1^ EDTA, 150 mmol l^-1^ NaCl, 1 mmol l^-1^ NaF, 1 mmol l^-1^ Na _3_VO_4_, 1% NP-40, 1% sodium deoxycholate, 1 mmol l^-1^ PMSF, and 1× HALT protease inhibitor cocktail (Thermo Fisher Scientific Inc.) at 24,000 rpm for 20 s each with 10 s intervals using the Polytron PT 1300D homogenizer (Kinematica AG, Lucerne, Switzerland). The homogenate was centrifuged at 10,000 ×g for 20 min at 4°C. The protein concentration in the supernatant obtained was determined according to the method of Bradford [42] and adjusted to 5 µg µl^-1^ with Laemmli buffer [43]. Samples were heated at 70°C for 15 min, and then kept at -80°C until analysis.

Proteins were separated by SDS-PAGE (8% acrylamide for resolving gel, 4% acrylamide for stacking gel) under conditions as described by Laemmli [43] using a vertical mini-slab apparatus (Bio-Rad Laboratories). Proteins were then electrophoretically transferred onto PVDF membranes using a transfer apparatus (Bio-Rad Laboratories). After transfer, membranes were blocked with 10% skim milk in TTBS (0.05% Tween 20 in Tris-buffered saline: 20 mmol l^-1^ Tris-HCl; 500 mmol l^-1^ NaCl, pH 7.6) for 1 h before being incubated overnight at 4°C with anti-NKCC antibody (T4, 1: 500 dilution) or pan-actin antibody (1:5000 dilution; Thermo Fisher Scientific Inc.). Antibodies were diluted in 1% bovine serum albumin in TTBS. Membranes were then rinsed and incubated with goat anti-mouse horseradish-peroxidase conjugated antibody (1:10,000 dilution; Santa Cruz Biotechnology, CA, USA) for 1 h at room temperature. Bands were visualized by chemiluminescence (Western Lightning^TM^, PerkinElmer Life Sciences, Boston, MA, USA) using X-ray film (Thermo Fisher Scientific Inc.) and were processed by a Kodak X-Omat 3000 RA processor (Kodak, Rochester, NY, USA). The films were scanned using CanonScan 4400F flatbed scanner in TIFF format at 300 dpi resolution. Densitometric quantification of band intensities were performed using ImageJ (version 1.40, NIH), calibrated with a calibrated 37 step reflection scanner scale (1″ x 8″; Stouffer no. R3705-1C). Results were presented as relative protein abundance of Nkcc normalized with actin.

The T4 antibodies developed by Christian Lytle (University of California Riverside, Riverside, CA) was obtained from the Developmental Studies Hybridoma Bank developed under the auspices of the National Institute of Child Health and Human Development and maintained by Department of Biological Sciences, The University of Iowa, Iowa City, IA. Although T4 was raised against human colonic NKCC, it reacts with not only the NKCC/Nkcc of mammalian and avian origins, but also those from elasmobranchs and teleosts [44,45]. Moreover, since there is 68% similarity between the epitope of T4 (MET-902 to SER-1212 of the carboxy-terminus of human NKCC) and the corresponding segment (ILE-798 to SER-1092) of Nkcc1b from *M. albus*, it is highly probable that T4 would recognize the *M. albus* Nkcc1b.

### Statistical analysis

Results were presented as means ± standard errors of the mean (S.E.M.). Differences between means were evaluated using one-way analysis of variance (ANOVA), followed by multiple comparisons of means by Tukey’s post-hoc test. Differences were regarded as statistically significant at *P* < 0.05.

## Results

### Ammonia concentrations in the brain and plasma

**Figure 1:**
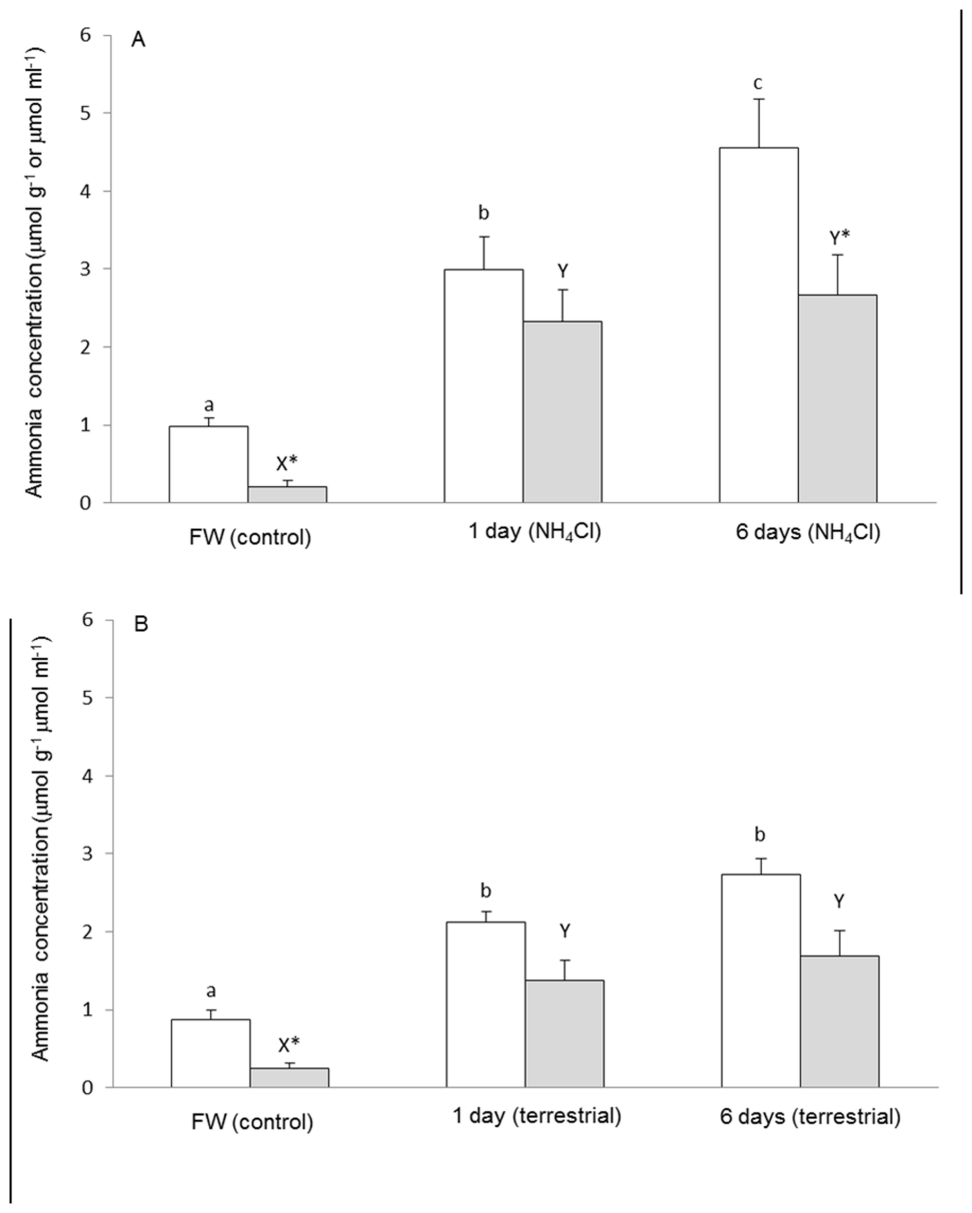
Concentrations of ammonia in the brain (µmol g^-1^; □) and plasma (µmol ml^-1^ or mmol l^-1^; ■) of *Monopterus* *albus* . Fish were kept in freshwater (FW; control) or (A) exposed to 50 mmol l^-1^ NH_4_Cl (pH 7.0) or (B) terrestrial conditions for 1 day or 6 days. Results represent means ± S.E.M. (N=5). Means not sharing the same letter (a, b, c or x, y) are significantly different (*P*<0.05). ^*^Significantly different from the value of the brain.

*Monopterus* *albus* can survive exposure to 50 mmol l^-1^ NH_4_Cl for 10 days without any overt deleterious effects. After 1 or 6 days of exposure to 50 mmol l^-1^ NH_4_Cl, there were significant increases in ammonia concentrations in the brain and plasma of *M. albus* (Figure 1A). The brain ammonia concentration reached 4.5 µmol g^-1^ on the sixth day of ammonia exposure. Exposure to terrestrial conditions for 1 or 6 days also led to significant increases, but at a slower rate, in the ammonia concentrations in the brain and plasma of *M. albus* (Figure 1B). After 6 days of exposure to terrestrial conditions, the brain ammonia concentration was only 2.7 µmol g^-1^, which was comparable to the ammonia concentration in the brain of fish exposed to NH_4_Cl for 1 day but lower (60%) than that of fish exposed to NH_4_Cl for a 6-day period.

### Nucleotide sequence, translated amino acid sequence and phylogenetic analysis of *nkcc*/Nkcc

**Figure 2:**
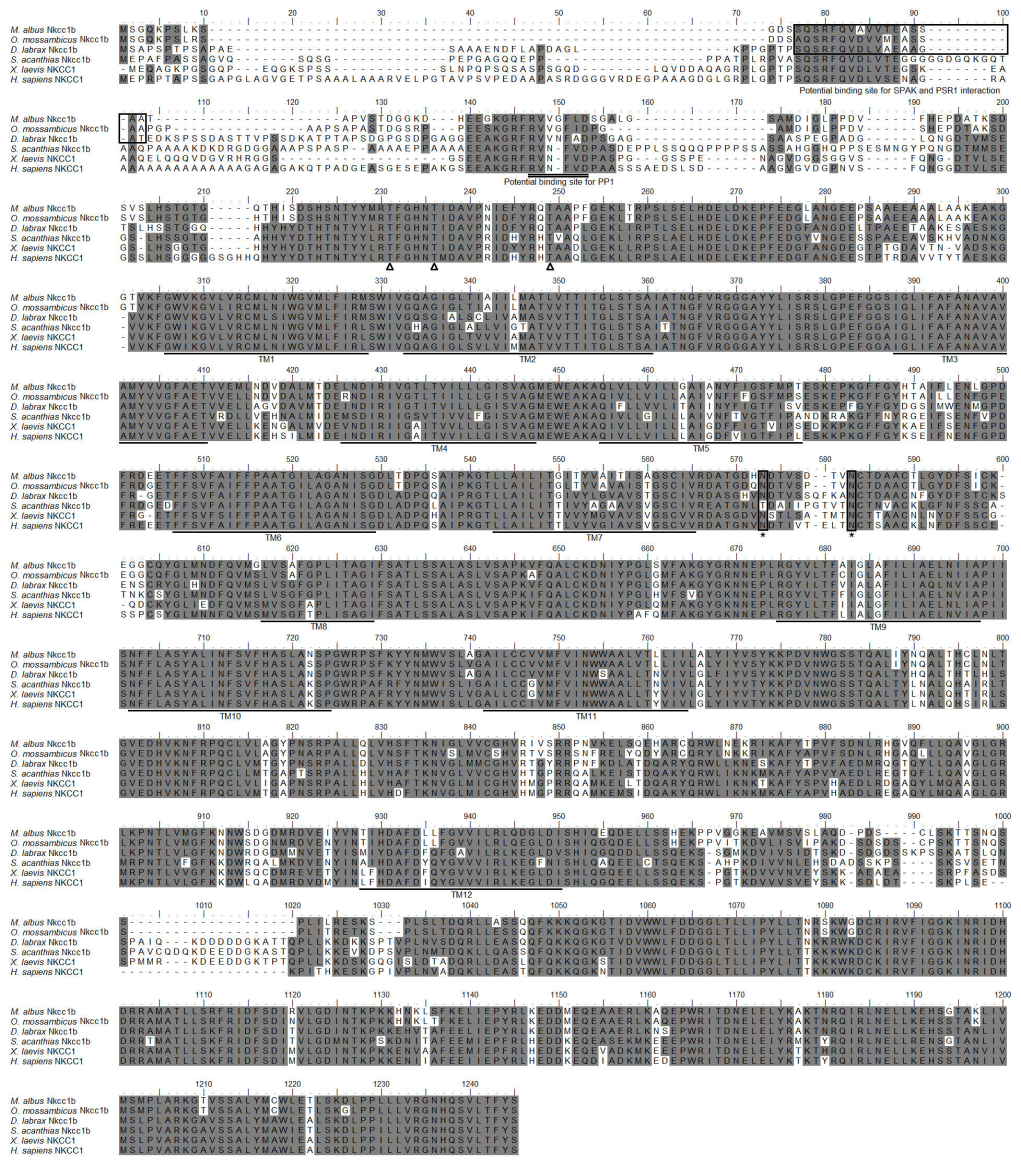
Molecular characterization of Na^+^:K^+^:2Cl^-^ cotransporter 1b (Nkcc1b) from the brain of *Monopterus* *albus* . A multiple sequence alignment of the Nkcc1b from the brain of *M. albus* with five other known Nkcc1/NKCC1 amino acid sequences from other animal species with Genbank accession numbers: *Oreochromis* *mossambicus* (AAR97732.1), *Dicentrarchus* *labrax* (ABB84251.1), *Squalus* *acanthias* (AAB60617.1), *Xenopus laevis* (ABN05233.1) and *Homo sapiens* (P55011.1). Shaded residues denote identical residues. Ste20-related proline-alanine-rich-kinase (SPAK) interaction site is indicated with boxes and protein phosphatase 1 (PP1) interaction site is double-underlined. Predicted phosphorylation sites are indicated by open triangles while potential *N-*glycosylation sites are indicated with boxes and ‘*’. The predicted transmembrane domains (TM) are underlined. The transmembrane domains of Nkcc1b of *M. albus* were predicted using MEMSATS & MEMSAT-SVA provided by PSIPRED protein structure prediction server.

**Figure 3:**
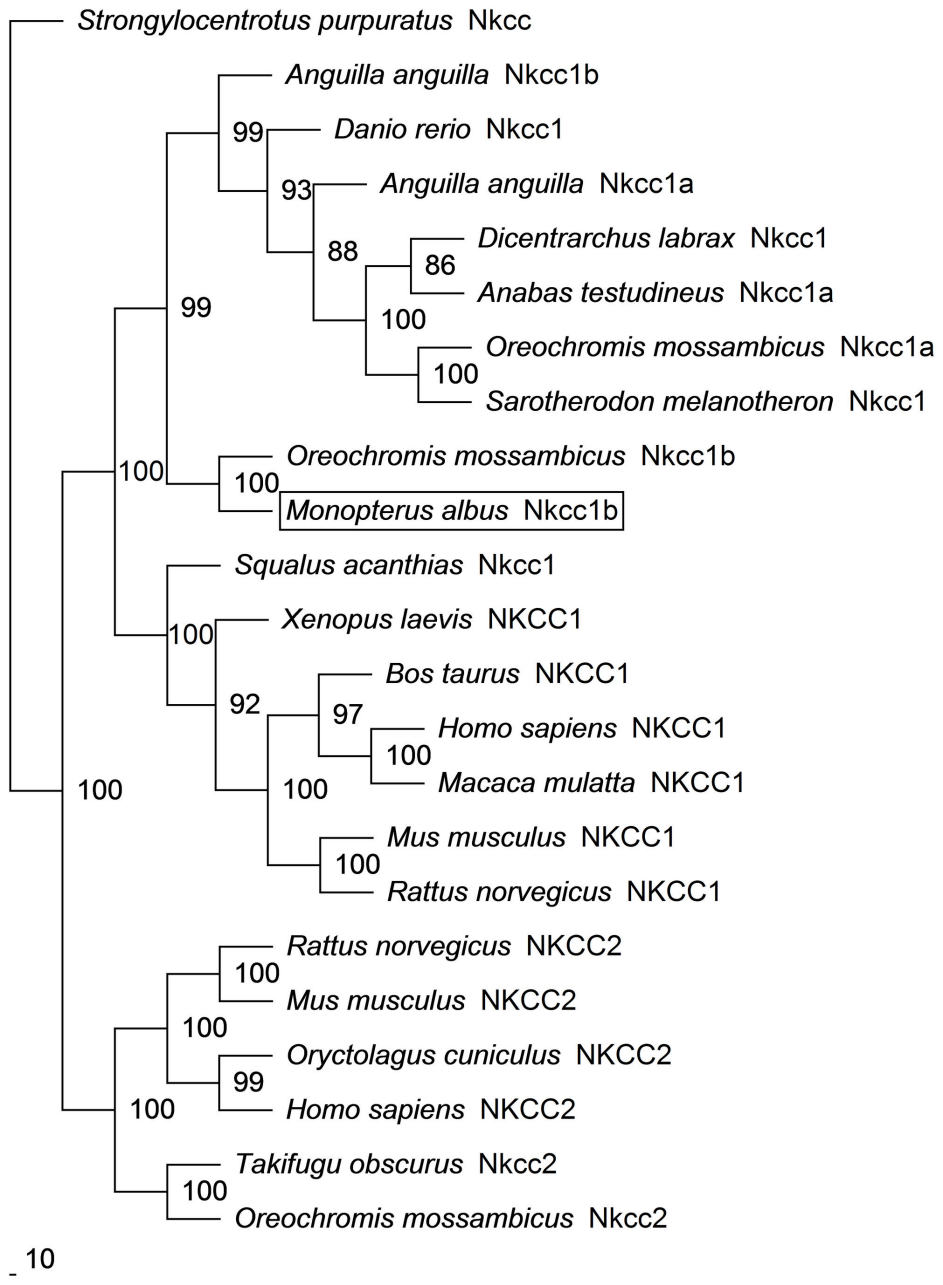
Phylogenetic analysis of Na^+^:K^+^:2Cl^-^ cotransporter 1b (Nkcc1b) from the brain of *Monopterus* *albus* . A phylogenetic tree to illustrate the relationship between Nkcc1b from the brain of *M. albus* and Nkcc/NKCC of selected vertebrate species. Numbers presented at each branch point represent bootstrap values from 100 replicates. *Strongylocentrotuspurpurtus* Nkcc is used as the outgroup for the phylogenetic tree for Nkcc.

The full cDNA coding sequence of *nkcc* from the brain of *M. albus* (GenBank accession number KC800686) comprised 3276 bp (Figure S1), coding for 1092 amino acids (Figure 2) with an estimated molecular mass of 119.6 kDa. It had an NH_2_-terminal sequence of approximately 183 amino acid residues, followed by 12 predicted transmembrane domains, and a COOH-terminal sequence (Figure 2). The putative Nkcc sequence had three potential phosphorylation sites and two *N*-glycosylation sites. An alignment of Nkcc of *M. albus* with those of two teleosts (sea bass and tilapia), shark, frog and human revealed that the NH_2_-terminal was the least conserved. The percentage similarities between Nkcc of *M. albus* and those of other teleosts were 71.4–90.6% for Nkcc1b, 69.0–71.1% for Nkcc1 or Nkcc1a and 51.2–53.0% for Nkcc2 (Table 1). A phylogenetic analysis provided additional support that Nkcc of *M. albus* is closer to teleost Nkcc1b than to teleost Nkcc1a or Nkcc2 (Figure 3). Based on these evidences, the Nkcc sequence found in the brain of *M. albus* was identified as Nkcc1b.

**Table 1:** The percentage similarity between the deduced amino acid sequence Na^+^:K^+^:2Cl^-^ cotransporter 1b (Nkcc1b) from the brain of *Monopterus* *albus* and Nkcc/NKCC sequences of other animal species obtained from GenBank (accession numbers in brackets).

Classification	Species	Similarity
Teleosts	*Oreochromis* *mossambicus* Nkcc1b (AAR97732.1)	90.6%
	*Anguilla* *anguilla* Nkcc1b (CAD31112.1)	71.4%
	*Salmo salar* Nkcc1a (NP_001117155.1)	71.1%
	*Anguilla* *anguilla* Nkcc1a (CAD31111.1)	71.0%
	*Sarotherodon* *melanotheron* Nkcc1 (ACY05529.1)	70.9%
	*Oreochromis* *mossambicus* Nkcc1a (AAR97731.1)	70.8%
	*Dicentrarchus* *labrax* Nkcc1 (ABB84251.1)	70.7%
	*Anabas* *testudineus* Nkcc1a (AFK29496.1)	69.2%
	*Danio rerio* Nkcc1 (NP_001157126.1)	69.0%
	*Oreochromis* *mossambicus* Nkcc2 (AAR97733.1)	53.0%
	*Takifugu* *obscurus* Nkcc2 (BAH20440.1)	51.2%
Elasmobranch	*Squalus* *acanthias* Nkcc1 (AAB60617.1)	62.1%
Amphibians	*Xenopus laevis* NKCC1 (ABN05233.1)	63.9%
Mammals	*Rattus norvegicus* NKCC1 (NP_113986.1)	62.3%
	*Bos taurus* NKCC1 (NP_777207.1)	62.8%
	*Mus musculus* NKCC1 (NP_033220.2)	62.6%
	*Homo sapiens* NKCC1 (P55011.1)	62.3%
	*Rattus norvegicus* NKCC2 (NP_001257547.1)	57.4%
	*Homo sapiens* NKCC2 (NP_000329.2)	57.4%
	*Mus musculus* NKCC2 (CAM17720.1)	57.0%

Sequences are arranged in a descending order of similarity.

### mRNA expression of *nkcc1b* in the brain

**Figure 4:**
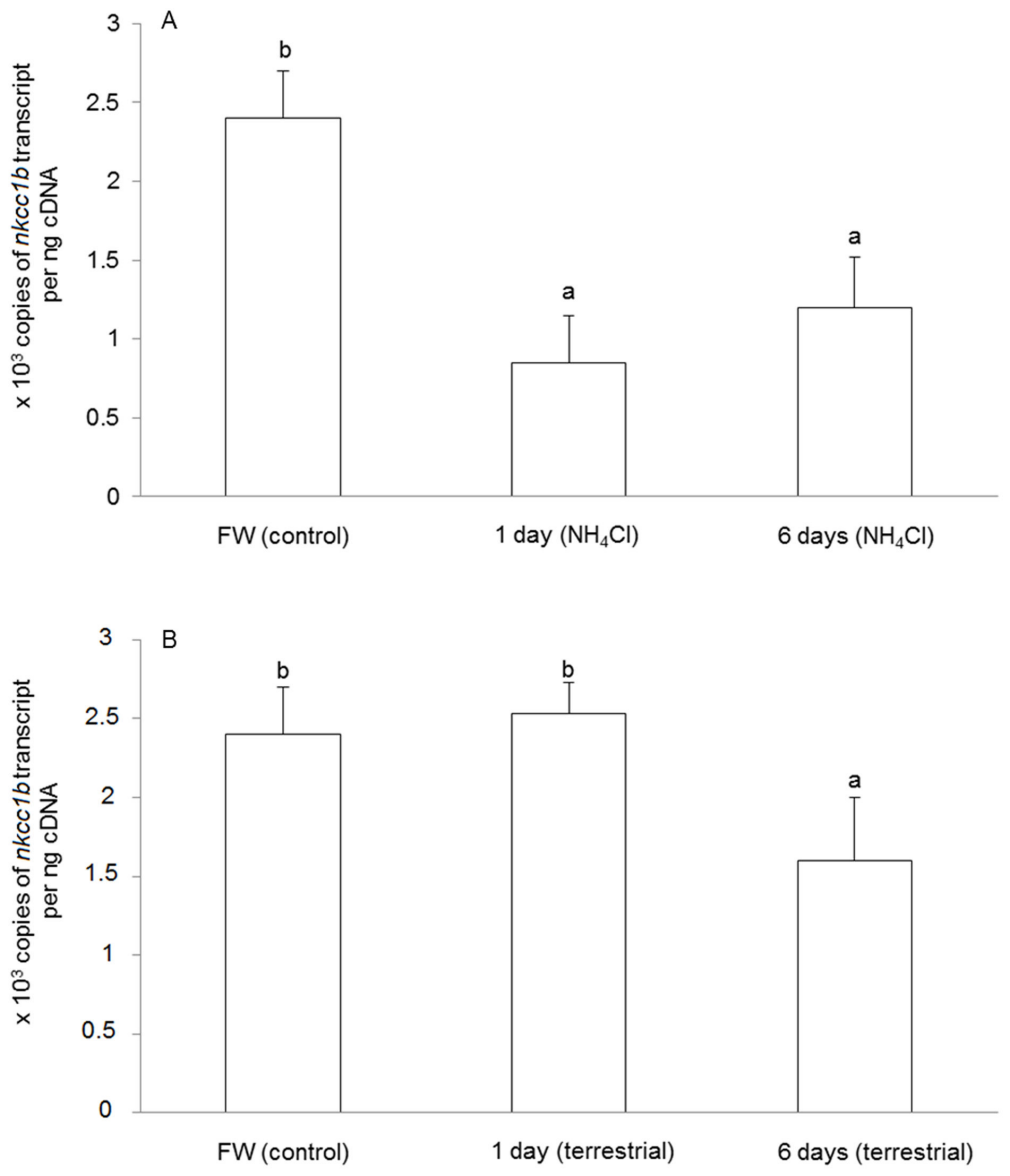
Effects of ammonia exposure or terrestrial exposure on *Na* ^*+*^ *:K* ^*+*^ *:2Cl* ^*-*^ *cotransporter 1b* (*nkcc1b*) mRNA expression in the brain of *Monopterus* *albus* . Absolute quantification (×10^3^ copies of transcript per ng of cDNA) of mRNA expression of *nkcc1b* in the brain of *M. albus* (A) kept in freshwater (FW; control) or exposed to 50 mmol l^-1^ NH_4_Cl for 1 day or 6 days, or (B) kept in FW (control) or exposed to terrestrial conditions for 1 day or 6 days. Results represent means + S.E.M. (N=5). Means not sharing the same letter are significantly different (*P*<0.05).

The mRNA expression of *nkcc1b* decreased significantly in the brain of *M. albus* after 1 day (by 61.1%) or 6 days (by 51.1%) of exposure to 50 mmol l^-1^NH_4_Cl as compared with the freshwater control (Figure 4A). Although there was no significant change in *nkcc1b* mRNA expression in the brain of *M. albus* exposed to terrestrial conditions for 1 day, the brain of fish exposed to terrestrial conditions for 6 days showed a significant decrease in the mRNA expression of *nkcc1b* as compared to that of the freshwater control (Figure 4B).

### Protein abundance of Nkcc1b in the brain

**Figure 5:**
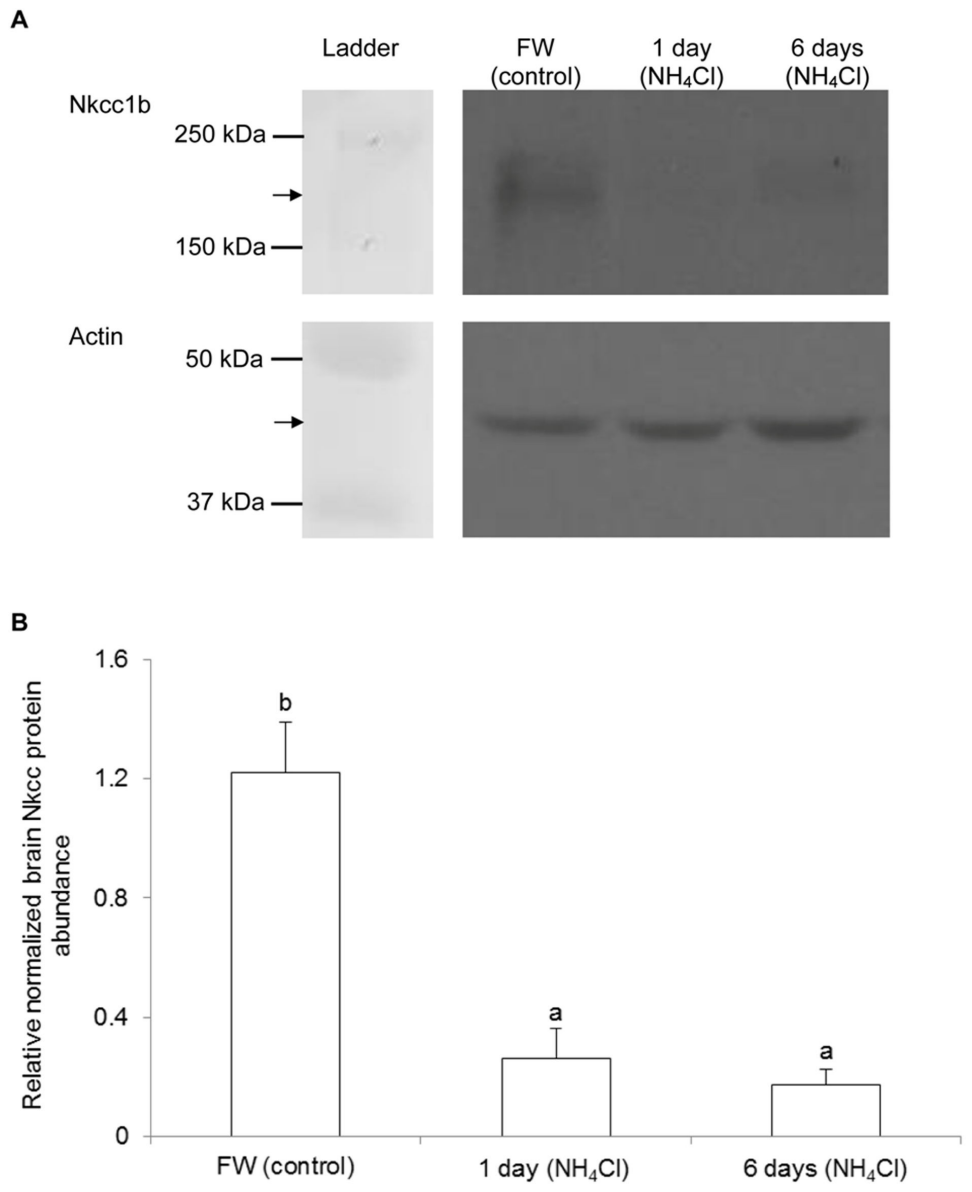
Effects of ammonia exposure on Na^+^:K^+^:2Cl^-^ cotransporter 1b (Nkcc1b) protein abundance in the brain of *Monopterus* *albus* . Protein abundance of Nkcc1b in the brain of *M. albus* kept in freshwater (FW; control) or exposed to 50 mmol l^-1^ NH_4_Cl for 1 day or 6 days. (A) An example of the immunoblots of Nkcc1b and actin. (B) The intensity of the Nkcc1b band normalized with respect to that of actin. Results represent mean + S.E.M. (N=3). Means not sharing the same letter are significantly different (*P*<0.05).

**Figure 6:**
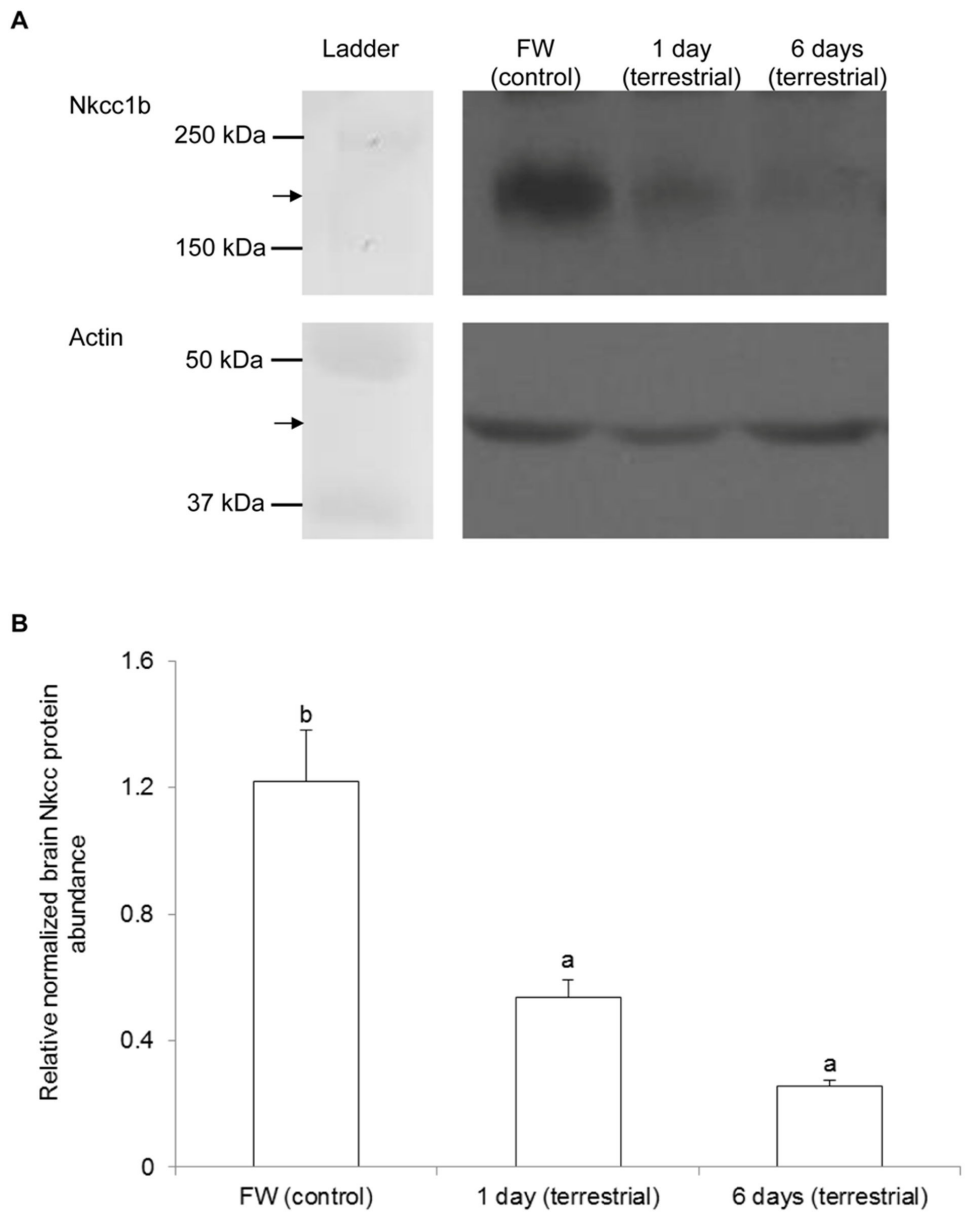
Effects of terrestrial exposure on Na^+^:K^+^:2Cl^-^ cotransporter 1b (Nkcc1b) protein abundance in the brain of *Monopterus* *albus* . Protein abundance of Nkcc1b in the brain of *M. albus* kept in freshwater (FW; control) or exposed to terrestrial conditions for 1 day or 6 days. (A) An example of the immunoblots of Nkcc1b and actin. (B) The intensity of the Nkcc1b band normalized with respect to that of actin. Results represent mean + S.E.M. (N=3). Means not sharing the same letter are significantly different (*P*<0.05).

Western blotting of Nkcc revealed the possibility of multiple bands, which presumably represented Nkcc at various glycosylation states spreading over a certain area of the gel, from the brain of *M. albus* kept in freshwater (control). There were significant decreases in the Nkcc band intensity, indicating decreases in the protein abundance of *nkcc1b* in the brain of *M. albus*, after 1 or 6 days of exposure to 50 mmol l^-1^ NH_4_Cl (Figure 5) or terrestrial conditions (Figure 6) as compared with the freshwater control.

## Discussion

NKCC1 is involved in ion transport across secretory and absorptive epithelia [29]. It is inactive in the basolateral membranes of secretory cells but can be activated through phosphorylation as a result of cell shrinkage or the presence of secretagogues [46,47]. The functional role of Nkcc in fish gills has been well established [45]. Two *nkcc1* (*nkcc1a* and *nkcc1b*) have been identified in the gills of the European eel. The mRNA expression of *nkcc1* is up-regulated in the gills of European eel, killifish, striped bass, tilapia and brackish medaka transferred from freshwater to seawater. Furthermore, the protein abundance of Nkcc increases in the gills of brown trout, tilapia, killifish, striped bass, salmonids and brackish medaka during seawater acclimation. Recently, Loong et al. [48] reported that both seawater acclimation and environmental ammonia exposure led to increases in the mRNA expression and the protein abundance of *nkcc1*/Nkcc1 in the gills of the climbing perch, *Anabas* *testudineus* , which has high environmental ammonia tolerance (~100 mmol l^-1^ NH_4_Cl at pH 7.0). Since there is a dearth of information on Nkcc in fish brains, especially concerning its relationship with brain ammonia toxicity and/or tolerance, results presented herein are novel.

### Molecular characterization of Nkcc1b from the brain of *M. albus*

Based on percentage identity and phylogenetic analysis, the full cDNA sequence of *nkcc* from the brain of *M. albus* was identified as *nkcc1b* (GenBank accession number KC800686). Darman and Forbush [49] demonstrated that at least three amino acid residues in NKCC would undergo phosphorylation upon activation, and our results indicated that Nkcc1b from the brain of *M. albus* contained these three phosphorylation sites (Thr231, Thr236 and Thr249). Studies on Nkcc from shark rectal gland have shown an increase in phosphorylation of serines and threonines in response to forskolin (a cyclic AMP agonist known to regulate NKCC) or hypertonic stress [46,50]. Moreover, like other NKCC/Nkcc, Nkcc1b from the brain of *M. albus* also possessed the consensus sites for *N*-linked glycosylation within a large hydrophilic loop between putative transmembrane domains 7 and 8 [51]. The presence of the phosphorylation and glycosylation sites in the *M. albus* Nkcc1b indicates that it can be regulated through post-translational modification in response to changes in conditions of the brain.

Piechotta et al. [52] identified a Ste20-related proline-alanine-rich kinase (SPAK) and oxidation stress response kinase 1 (OSR1) interaction site in the N terminus of NKCC, and suggested that sea bass Nkcc1 may be activated by stress kinases in response to salinity changes [53]. Both OSR1 and SPAK interaction sites are present in the N terminus of Nkcc1b from the brain of *M. albus*, indicating that it could be activated through phosphorylation and/or glycosylation by osmotic and/or oxidative stresses. This information is relevant to understanding why defense against ammonia toxicity in the brain of *M. albus* would involve Nkcc1b. In human, brain edema is a critical component of hepatic encephalopathy associated with acute liver failure [3,4]. Brain edema appears to be principally due to astrocyte swelling (cytotoxic edema), and a major factor responsible for astrocyte swelling is believed to be ammonia [4,5]. Since NH_4_^+^ can substitute K^+^ to activate Nkcc and Nkcc of *M. albus* could also respond to osmotic stress through OSR1 and SPAK, it is logical to deduce that a suppression of *nkcc*/Nkcc expression in the brain would contribute to high brain ammonia tolerance. Furthermore, acute ammonia intoxication can activate *N*-methyl-ᴅ-aspartate-type glutamate receptors [6,7], and excessive activation of these receptors in turn leads to oxidative stress, neuronal degeneration and death in mammals [8]. Ammonia can also affect directly the intracellular NO and/or Ca^2+^ concentrations, increases in which can lead to increased production of free radicals [54]. Indeed, it has been established that fish brain also experiences ammonia-induced oxidative stress. Exposure of the mudskipper, *Boleophthalmus* *boddarti* , to 8 mmol l^-1^ NH_4_Cl for 12 or 24 h leads to the accumulation of carbonyl proteins, elevation in oxidized glutathione content and oxidized: reduced glutathione ratio, decreases in activities of glutathione reductase and catalase, and an increase in the activity of superoxide dismutase in its brain [55]. Hence, to achieve high brain ammonia tolerance, the expression of *nkcc*/Nkcc, which could respond to oxidative stress through OSR1 and SPAK, must be suppressed in the brain of *M. albus* confronted with ammonia toxicity.

### Down-regulation of *nkcc1b* mRNA and Nkcc1b protein abundance in the brain of *M. albus* exposed to environmental ammonia and its implications

When exposed to 50 mmol l^-1^ NH_4_Cl, both NH_3_ and NH_4_^+^ gradients are driving ammonia into the body of *M. albus*. Therefore, the fish is confronted simultaneously with the impediment of endogenous ammonia excretion and exogenous ammonia infiltration. Once endogenous or exogenous ammonia enters the blood, it would exert toxic effects on all cell types, particularly those in the brain. Bumetanide-inhibitable NKCC expression has been demonstrated in endothelial cells in bovine [56] and rat brain [57], and it functions to maintain a low steady state concentration of K^+^ in the brain interstitial fluid for normal neuronal activity. It has been suggested that NH_4_^+^ can substitute for K^+^ and permeate the blood-brain barrier through NKCC [25]. Hence, it would appear that a down-regulation of endothelial NKCC would help to ameliorate brain ammonia toxicity, because, in human, encephalopathy develops at a brain ammonia concentration of >1 µmol g^-1^ [3]. However, since the blood-brain barrier has greater permeability to NH_3_ than to NH_4_^+^ [24], it would be ineffective to ameliorate ammonia toxicity through a down-regulation of Nkcc1b to reduce the permeation of NH_4_^+^ at the blood-brain barrier. In fact, the permeation of ammonia through the blood-brain barrier of *M. albus* is confirmed by the accumulation of ammonia to high concentrations in the brain of fish exposed to ammonia or terrestrial conditions for 6 days. Thus, it becomes apparent that the down-regulation of *nkcc1b* mRNA expression and Nkcc1b protein abundance in the brain of *M. albus* exposed to environmental ammonia represented a response of neurons and/or other cell types to combat high concentrations of extracellular ammonia in the brain.

NKCC1 expression has been detected in cell bodies and dendrites of pyramidal neurons in cerebral cortex of rats, and it facilitates the accumulation of Cl^-^ in neurons [58]. Kelly and Rose [58] investigated mechanisms of cellular NH_4_^+^ influx in hippocampal slices by measuring acute NH_4_^+^/NH_3_-induced changes in intracellular pH and Na^+^ concentration. Their results suggest that, following a rapid elevation of plasma ammonia, NH_4_^+^ influx occurs mainly through Na^+^/K^+^-ATPase in neurons, whereas NKCC, working in conjunction with Na^+^/K^+^-ATPase, mediates NH_4_^+^ influx in astrocytes [59]. Swelling of astrocytes represents the most prominent neuropathological abnormality in acute liver failure [60]. Ammonia has been shown to induce swelling of astrocytes *in vivo* [61], and *in vitro* [62]. It also activates oxidation/nitration and increases total NKCC1 protein expression in cultured astrocytes. When treated with bumetanide, an NKCC inhibitor, cultured astrocytes exposed to ammonia significantly reduced ammonia-induced swelling [27]. Administration of antioxidants and cycloheximide also significantly reduces the ammonia-induced increase in NKCC1 activity [27]. Ammonia-induced increase in oxidative and/or nitrosative stress probably enhances NKCC phosphorylation through the activation of mitogen-activated protein kinases, and up-regulates NKCC1 protein expression through the activation of transcription factor NF-κB, resulting in increased NKCC activity. Similarly, trauma increases the phosphorylation (activation) of NKCC1, while inhibition of mitogen-activated protein kinases and oxidative/nitrosative stress diminishes the trauma-induced NKCC1 phosphorylation as well as its activity [28]. Bumetanide significantly reduces the trauma-induced astrocyte swelling by 61%, and silencing NKCC1 with siRNA leads to a reduction in trauma-induced NKCC1 activity and cell swelling. Hence, it can be concluded that up-regulation of NKCC/NKCC expression plays an important role in the development of astrocyte swelling/brain edema in mammals.

By contrast, we report for the first time that ammonia exposure resulted in down-regulation of *nkcc1b* mRNA expression and Nkcc1b protein abundance in the brain of *M. albus*. It is probable that *M. albus* was able to prevent swelling of brain cells and avoid brain edema through regulating Nkcc1b activity, and such an ability contributed in part to its high brain ammonia tolerance. Taken together, our results are in agreement with the proposition that blocking NKCC1 activity may represent a useful therapeutic strategy for cytotoxic brain edema [28]. Of note, NKCC1 is also known to promote water transport across cell membranes [51]. Studies performed on cultured pigmented epithelial cells from the ciliary body of the fetal human eye transfected with NKCC1 reveal that NKCC1 works both as a water channel allowing passive water flux and as a water pump that transports water across apical membranes regardless of osmotic gradients [63]. Recent studies have demonstrated that NKCC1 transports 500 water molecules for each cycle of cation-chloride transport [64], which is comparable to that of aquaporins. Therefore, it is probable that reduction in the mRNA expression and protein abundance of *nkcc1b*/Nkcc1b in the brain of *M. albus* exposed to ammonia could directly ameliorate the severity of ammonia-induced cell swelling and brain edema.

### Terrestrial exposure took a longer period before leading to down-regulation of *nkcc1b* mRNA expression and Nkcc1b protein abundance in the brain of *M. albus*

When *M. albus* was exposed to terrestrial conditions, ammonia accumulated in the body, but the rate of ammonia accumulation was relatively slow compared with fish exposed to 50 mmol l^-1^ NH_4_Cl. Thus, the lack of significant changes in the mRNA expression of *nkcc1b* in the brain of fish exposed to terrestrial conditions for 1 day can be related to the relatively low concentration of ammonia accumulated therein. However, 1 day of terrestrial exposure resulted in a significant decrease in the brain Nkcc1b protein abundance, indicating that regulation of Nkcc activity could be achieved through protein synthesis and/or degradation. For *M. albus* exposed to terrestrial conditions for 6 days, the brain ammonia concentration reached a level comparable to that of fish exposed to 50 mmol l^-1^ NH_4_Cl for 1 day, which was apparently high enough to induce significant decreases in the mRNA expression of *nkcc1b* and protein abundance of Nkcc1b in the brain of *M. albus*.

### Perspective

Air-breathing fishes, particularly amphibious ones, are equipped with various strategies to ameliorate ammonia toxicity during emersion or ammonia exposure. The responses of air-breathing fishes to ameliorate ammonia toxicity are many and varied, determined by the behavior of the fish and the nature of the environment in which it lives [18,19,20]. Active ammonia excretion, operating in conjunction with lowering of ambient pH and reduction in branchial and/or cutaneous NH_3_ permeability, as in the case of the giant mudskipper, *Periophthalmodon* *schlosseri* [65,66,67,68], and the climbing perch, *A* *. testudineus* [69], is theoretically the most effective strategy to maintain low internal (plasma and tissue) ammonia concentrations. Recent reports on *A* *. testudineus* , which can survive a progressive acclimation from freshwater to seawater and exposure to high concentration of environmental ammonia in freshwater, revealed that both active salt excretion during seawater acclimation and active NH_4_^+^ excretion during ammonia exposure (in freshwater) could involve similar transport mechanisms (Nkcc1, cystic fibrosis transmembrane conductance regulator and Na^+^/K^+^-ATPase) but two different types of Na^+^/K^+^-ATPase-immunoreactive cells in its gills [48,70,71]. In comparison, *M. albus* has degenerate gills and is incapable of active ammonia excretion. Therefore, it is logical for *M. albus* to develop the ability to tolerate high concentrations of ammonia at the cellular level, especially in the brain. Our results suggest for the first time that the ability to down-regulate the mRNA expression of *nkcc1b* and protein abundance of Nkcc1b in the brain could be one of the contributing factors to the extraordinarily high brain ammonia tolerance in *M. albus*. Efforts are being made in our laboratory to determine the localization of Nkcc1b in the brain of *M. albus*, and its functional relationship with other transporters, e.g. Na^+^/K^+^-ATPase. Since NH_4_^+^ can enter brain cells through Nkcc1b, it is probable that, similar to the gills of *A* *. testudineus* [70], the brain of *M. albus* may express a Na^+^/K^+^-ATPase α-subunit isoform that can better differentiate K^+^ from NH_4_^+^ so as to maintain intracellular K^+^ homeostasis when the brain is confronted with ammonia toxicity. The confirmation of this hypothesis awaits future studies.

## Supporting Information

Figure S1 **Nucleotide sequence (GenBank accession number KC800686) and translated amino acid sequence of the full coding region of Na^+^:K^+^:2Cl^-^ cotransporter 1b from the brain *Monopterus* *albus* .** The start codon is indicated by the first ATG, while the stop codon is indicated by an asterisk.
(TIF)
