# Supplementary figures and images for: High Brain Ammonia Tolerance and Down-Regulation of Na+:K+:2Cl- Cotransporter 1b mRNA and Protein Expression in the Brain of the Swamp Eel, Monopterus albus, Exposed to Environmental Ammonia or Terrestrial Conditions
Source: PLoS One. 2013 Sep 19;8(9):e69512. doi: 10.1371/journal.pone.0069512 (PMC3777983; doi:10.1371/journal.pone.0069512)

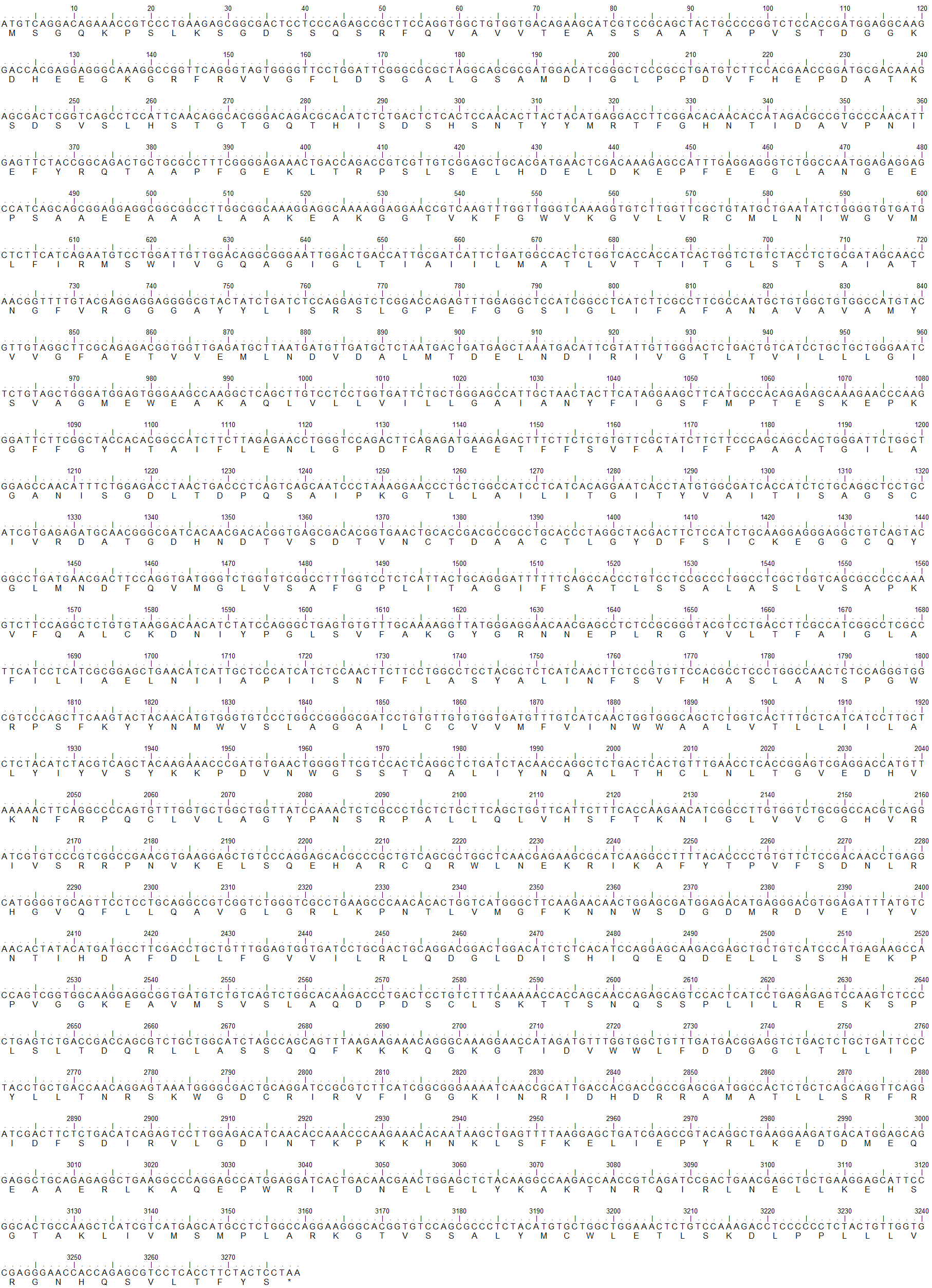

Supplement: Figure S1 — Nucleotide sequence (GenBank accession number KC800686) and translated amino acid sequence of the full coding region of Na+:K+:2Cl- cotransporter 1b from the brain Monopterus albus . The start codon is indicated by the first ATG, while the stop codon is indicated by an asterisk. (TIF) [file pone.0069512.s001.tif]
